# A Remote Adapted Physical Activity Intervention for Women With Breast Cancer and Severe Depressive or Anxiety Symptoms: Series of N-of-1 Trials With Ecological Momentary Assessment

**DOI:** 10.32872/cpe.14851

**Published:** 2025-08-29

**Authors:** Johan Caudroit, Samuel St-Amour, Josyanne Lapointe, Ahmed Jerôme Romain, Alain Steve Comtois, Guillaume Chevance, Paquito Bernard

**Affiliations:** 1 Laboratoire sur les vulnérabilités et l’innovation dans le Sport, Université Claude Bernard Lyon 1, Villeurbanne, France; 2 Health Sciences Department, Université du Québec à Rimouski, Rimouski, QC, Canada; 3 Physical Activity Sciences Department, Université du Québec à Montréal, Montreal, QC, Canada; 4 Research Centre, University Institute of Mental Health in Montreal, Montreal, QC, Canada; 5 School of Kinesiology and Physical Activity Sciences, Faculty of Medicine, Université de Montréal, Montreal, QC, Canada; 6 EHESP, Inserm, IRSET (Institut de Recherche en Santé, Environnement et Travail) – UMR_S, 1085, Université de Rennes, Rennes, France; Philipps-University of Marburg, Marburg, Germany

**Keywords:** psychological distress, cancer, physical exercise, tele-health, single case experimental study

## Abstract

**Background:**

Women with breast cancer live with the burden of the disease, its treatment, and the psychosocial consequences of illness, often contributing to the experience of psychological distress. At this end, physical activity (PA) is an evidence-based strategy to decrease depressive and anxiety symptoms. However, no study has yet investigated how those psychological symptoms fluctuate and vary during a PA intervention at the individual level, especially for individuals with severe psychological distress. Thus, the aim of the present study was to examine the short-term effects of a 12-week remote PA intervention on daily level of depressive and anxiety symptoms among women with breast cancer and severe depressive or anxiety symptoms.

**Method:**

A N-of-1 study followed an ABA’ design was conducted. Each A phase (2-week) represents pre- and post-intervention phase and B phase (12-week) represents the intervention phase. For the whole 16 weeks, participants received a daily prompt to report their depressive and anxiety levels. The intervention combined two to three (un)supervised remote PA sessions per week coupled with weekly text messages.

**Results:**

Sixteen participants completed the intervention. A significant decrease of depressive and anxiety symptoms was found for nine and seven participants, respectively. Different temporal patterns of depressive and anxiety were observed during and after the intervention. Interestingly, the impact of PA intervention was generally not immediate and gradual.

**Conclusion:**

This study supports the utility of remote PA intervention to improve depressive and anxiety symptoms in women with breast cancer and poor mental health.

## Highlights

- A remote physical activity intervention is beneficial for adults experiencing severe psychological distress.
- N-of-1 coupled with EMA is a solid method to observe the fluctuation of mental health symptoms.
- Physical activity benefits on mental health symptoms are not immediate but gradual.

Poor mental health is a major clinical issue during and post-breast cancer treatment (Caruso et al., 2017; Kissane, 2014; Niedzwiedz et al., 2019). Previous meta-analyses have reported that the prevalence of anxiety and depressive symptoms among women with breast cancer (BC) reached 44.2% and 28.9% for moderate levels and 20% and 13.2% for severe level, respectively (Hashemi et al., 2020; Pilevarzadeh et al., 2019). Severe depressive or anxiety symptoms are associated with higher risk of mortality and cancer recurrence (Wang et al., 2020) but also with lower cancer treatments adherence, higher health related costs and impaired quality of life (Mausbach et al., 2015, 2018; Mitchell et al., 2013).

Regarding treatment options, psychological therapies and antidepressants can decrease the symptoms' intensity among women with a BC and moderate levels of depressive and anxiety symptoms. However, the effectiveness of these interventions is very modest (Vita et al., 2023; Xiao et al., 2017) and, in most cases, women with severe depressive and anxiety symptoms were excluded from clinical trials (Carvalho et al., 2014). Therefore, new interventional strategies must be explored and applied to women with BC and moderate to severe depressive and anxiety symptoms.

At this end, high level of evidence revealed that physical activity (PA) interventions are effective treatments to help adults with moderate or severe depressive or anxiety disorders (Ravindran et al., 2016). For BC, the American College of Sport Medicine (ACSM)’s guideline highlighted that PA interventions are an evidence-based strategy to decrease the risk of depressive or anxiety symptoms (Campbell et al., 2019). Meta-analyses including more than thirty randomized controlled trials, suggested that PA interventions decrease the depressive and anxiety symptoms during and after BC treatment (Carayol et al., 2013, 2015). However, none of these trials included women with a severe level of depression or anxiety (Bernard & Carayol, 2015). One reason is that designing an adapted PA intervention for those women is very challenging because the presence of depressive and anxiety symptoms decreases the PA engagement and adherence rate (Bullard et al., 2019). So, it is crucial to individualize and personalize the PA intervention, according to preferred PA modalities in women with BC (Schmitz et al., 2019) and in using behavioral change techniques during the intervention.

Furthermore, the daily fluctuations of those symptoms are complex to observe with a simple pre- and post-intervention measurement (Bentley et al., 2019). An appropriate method, such as single case experimental study (or N-of-1) is necessary to examine the evolution of depressive and anxiety symptoms during the intervention (Bentley et al., 2019). N-of-1 has received an increasing interest in oncology (Sequeira et al., 2023), PA (Lapointe et al., 2023), and mental health research (St-Amour et al., 2024). This design consists in collecting data regularly form participants as they progress through different phases of the study, i.e., the first phase measures the baseline of the interest variables and the subsequent phase serve to introduce the intervention. The data from the intervention phase are compared to the baseline phase, providing strong internal validity for each participant. In addition, conduction N-of-1 combined with Ecological Momentary Assessment (EMA) is a solid methodological approach to examine the effects of a clinical intervention on depressive and anxiety symptoms at individual level. EMA has been recommended to improve the ecological validity and reduce recall bias (St-Amour et al., 2024).

The goal of the present study was to examine the short-term effects of a 12-week supervised adapted PA intervention on daily levels of depressive and anxiety symptoms among women with BC and severe depressive or anxiety symptoms. We hypothesize that (1) daily level of depressive and anxiety symptoms will decrease during the intervention and (2) intervention effects were maintained 2 weeks after its end.

## Method

This research protocol has been approved by the Ethics Boards of the *Eastern Montreal Integrated University Health and Social Services Centre* (2022-4319). This manuscript was written according to the *Single-Case Reporting Guideline in BEhavioural Interventions* (SCRIBE; Tate et al., 2016). The SCRIBE checklist is provided in the Supplementary Materials (Bernard, 2025S).

### Study Design

This N-of-1 study followed an A-B-A design (representing the three phases of the study) and lasted 16 weeks. No randomization nor blinding were used due to the nature of the study. Each A phase (2-week each) represents the pre- and post-intervention baseline measures, and the B phase (12-week) represents the intervention phase. For the whole 16 weeks, participants received a daily EMA prompt to report their depressive and anxiety levels. The intervention (B phase) included two to three (un)supervised remote PA sessions per week coupled with motivational and educational text messages.

### Recruitment

Participants were recruited from Maisonneuve Rosemont or Santa Cabrini Hospitals (Montreal, Canada). Flyers, outlined the study aims, inclusion criteria and contact information for the research coordinator, were distributed through psycho-oncology consultations and shared to patients on the psycho-oncology unit waitlist and on the oncology center website and social networks. Potential participants were screened by telephone and included if they: 1) were diagnosed with a non-metastatic BC and currently completing treatment or completed treatment at least three months prior; 2) reported a high level of self-reported depressive (Patient Health Questionnaire score ≥ 15) or anxiety symptoms (General Anxiety Disorder’s questionnaire score ≥ 15); 3) were aged 18 to 65 years; 4) were considered inactive (less than 150 minutes per week or Godin’s questionnaire score < 23; Amireault et al., 2015); 5) possessed a smartphone. Participants were excluded if they: 1) reported a psychotic or schizophrenia disorder diagnostic; 2) answered positively to the PA Readiness Questionnaire for Everyone; 3) received a weekly psychological treatment from a clinician; 4) reported a major functional or physical disability; 5) were unable to provide consent. The consent form was sent via e-mail before the initial evaluation. Participants were rewarded $150 CA upon study completion.

### Physical Activity Intervention

The intervention combined a remote adapted PA intervention, supervised by a kinesiologist, coupled with weekly text messages for 12 weeks. The characteristics of this intervention were based on the ACSM recommendations, identified behavioral change techniques and PA preferences in interventional context among women with BC (Caudroit, Chevance, et al., 2025). The detailed description of the intervention development and content have been previously published (Caudroit, Lapointe, et al., 2025). This intervention has been found as feasible, acceptable, and associated with high adherence rates (Caudroit, Lapointe, et al., 2025).

Participants had three supervised remote PA sessions from week three to six. From week seven to 14, participants had two supervised remote PA sessions, with the choice between supervised or unsupervised sessions for the third weekly session. The supervised PA sessions included: 1) combination of behavioral change techniques and aerobic exercise with resistance or yoga exercises (participants’ choice); 2) the durations of sessions ranged from 30 (weeks 3-6) to 60 minutes (weeks 7-14) but actual durations were adaptable based on participants’ self-reported fatigue level; 3) the effort intensity was selected by the participant, guided by the proposed intensity of the day provided by the kinesiologist, explained through the rate of perceived exertion, progressively increasing, and varying between 1 and 8 (different for aerobics, resistance, yoga) on a scale of 10 during the 14 weeks. The unsupervised PA sessions could be based on previous supervised sessions or personalized after discussions with the participant (e.g., 2 weekly walks of 30 minutes).

### Measures

Online questionnaires and EMA were used to measure our variables of interest during the phases of our study. In line with the SCRIBE, we used validated questionnaires to describe participants demographic characteristics and clinical features (Tate et al., 2016). A research-oriented app was installed (EthicaData) on participants’ smartphones to obtain self-measured daily rating of depressive and anxiety symptoms.

### Questionnaires

**Figure 1:**
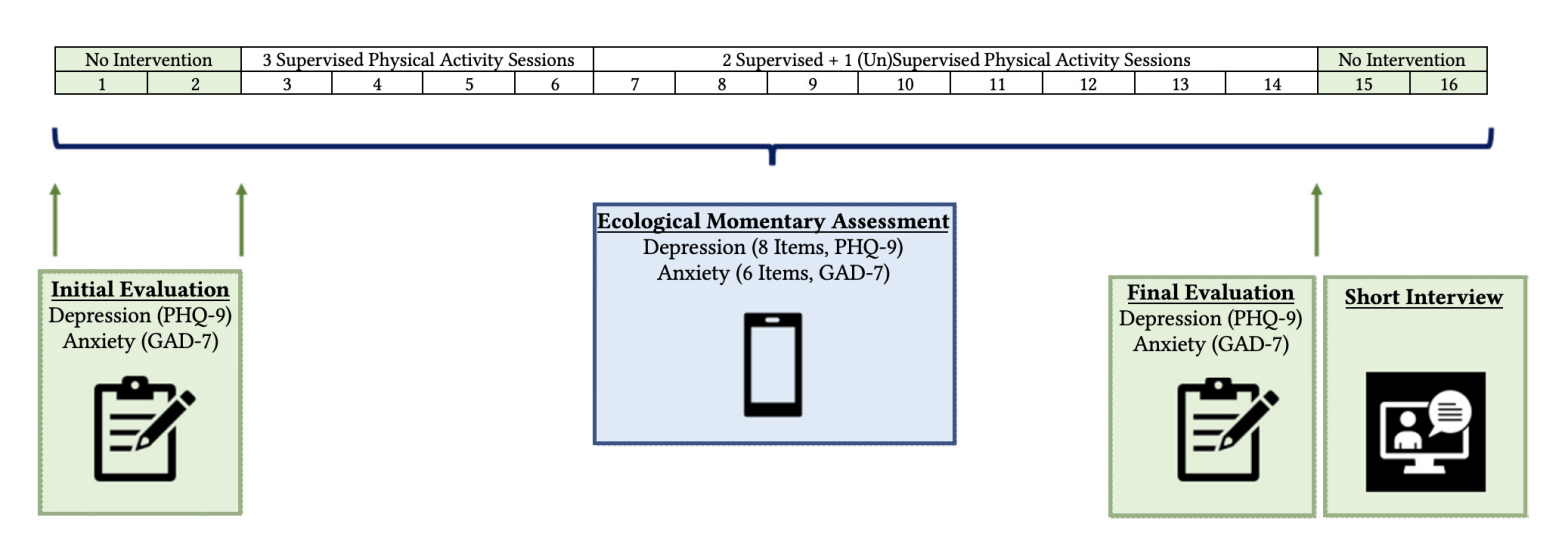
Study Design

Before the intervention, online questionnaires collected information on age, educational achievements, marital status, income, number of children, date of diagnosis, treatments, current medications, and history of cancer. According to the SCRIBE recommendations, generalization measures were also performed to increase the external validity of the study. Thus, GAD-7 and PHQ-9 were filled by participants three times during the study (details in Figure 1; Tate et al., 2016). The respective validated thresholds of these scales were used to describe the individual scores (see details in the Supplementary Materials [Bernard, 2025S]). The Patient Health Questionnaire (PHQ-9) is a screening instrument with nine items, developed to assess the severity of depression (Kroenke et al., 2001). For each item, the respondent is asked to rate how often each symptom occurred over the last two weeks, on a Likert scale ranging from 0 “not at all” to 3 “nearly every day”. The sum score (range to 0 to 27) indicates the degree of depression with score of ≥ 15 representing severe levels of depression (Howell et al., 2015). The Generalized Anxiety Disorder (GAD-7) is a one-dimensional instrument created to detect symptoms of generalized anxiety disorders (Spitzer et al., 2006). Core symptoms within the past two weeks were queried with seven items on a four-point Likert scale rated from 0 (not at all) to 3 (nearly every day). The total GAD-7 score can range from 0 to 21 and a score ≥ 15 represents severe anxiety symptoms levels (Howell et al., 2015). PHQ-9 and GAD-7 are both recommended questionnaires by the Canadian Association of Psychosocial Oncology (Howell et al., 2015).

### Daily Assessments

Every day during the Phase B, participants rated the severity of depressive and anxiety symptoms using a 0-100 visual analog slider (0: not at all – 100: as much as possible). Participants rated the severity of depression symptoms with 8 adapted items from the Patient Health Questionnaire (PHQ-9, Kroenke et al., 2001). The severity of anxiety symptoms was measured with 6 adapted items from the GAD-7 (Spitzer et al., 2006). All EMA items are presented in Table 1.

**Table 1:** Ecological Momentary Assessment Items

Variable / Item
Depressive symptoms
Today, I feel depressed
Today, I feel guilty
Today, I had difficulties in concentrating
Today, I feel tired
Today, I feel like I am in slow motion
Today, I have a good appetite
Today, I felt hopeless
Today, I have little interest or pleasure in what I am doing
Anxiety symptoms
Today, I have a feeling of fear
Today, I feel angry
Today, I feel worried
Today, I feel restless
Today, I feel irritable
Today, I am feeling muscle tension

### Internal Validity

Single case experimental studies may be subject to rival hypotheses that can explain changes in the dependent variables like maturation, question-behavioral effect, and other external factors (St-Amour et al., 2024). To ensure the internal validity (i.e., the observed change is attributable to the intervention rather than other factors), participants were questioned at the end of Phase B about important event having occurred in their life during the phase they just finished that could have a prolonged (positive or negative) impact on their mental health symptoms.

### Statistical Analyses

A piecewise linear regression has been performed for each participant. This analysis is particularly well-suited for time series with Gaussian or Poisson distribution (Lapointe et al., 2023; Wilbert, 2021). We considered the auto-correlation between symptoms data only for models with a Gaussian distribution because it was implemented only for the latter in the scan package (Wilbert, 2021). Through those analyses, we compared the daily depression and anxiety level during and after the intervention to baseline measures. Each piecewise regression was carried out to examine the level effects (i.e., the difference between the mean of symptoms in Phase A with Phase B and A’, divided by the standard deviation of the residuals) and test the slope effects (i.e., the continuous change of symptom levels due to the PA intervention). Two models were systematically compared by including or not the trend. The model with the highest *R*^2^ was finally selected for each dependent variable.

### Transparency and Openness

The study protocol was registered a priori with OSF registries (see Supplementary Materials [Bernard et al., 2022S]). Analyses and graphics have been performed with R 4.3 and ggplot2, and scan packages. Data, open materials, and R scripts are available online (Bernard, 2024S-a).

## Results

### Participants Characteristics

Between 2022 May and 2023 June, 18 participants (10 during treatment and eight post-treatment) were included. Baseline sociodemographic, health and cancer-related information, and PA adherence sessions rates are in Table 2. Two participants were dropped during this study. Some participants postponed the beginning of their intervention phase due to events out of their control making some A phases longer for some than others. Regarding scores on PHQ-9 and GAD-7, all participants experienced severe psychological distress (score ≥ 15 on, at least, one scale) with 13 patients who had severe depressive disorders, one who had severe anxiety disorders and four who had severe depressive and anxiety disorders. The EMA adherence rates were ranged from 48% to 88%, with eight participants with a rate above 70%.

**Table 2:** Study Participant Characteristics

ID	Age	Marital status	Education	Working status	Incomes (k$)	BMI	Cancer stage	In treatment^a^	Smoking	GAD7	PHQ9	Adherence
id1	44	Single	Coll	SL	40-60	23	I	Yes	No	13	17	62
id2	43	Single	Coll	SL	40-60	DK	III	No	No	15	17	100
id3	59	Married	Coll	FTW	>80	25	II	Yes	No	14	16	85
id4	29	Married	High S	SL	>80	48	IV	Yes	No	10	18	97
id5	38	Married	Univ	SL	20-40	30	II	Yes	No	12	15	94
id6	44	Married	Univ	SL	>80	27	I	Yes	No	12	15	88
id7	61	Single	Univ	PTW	40-60	14	I	Yes	No	13	21	82
id8	45	Single	Coll	FTW	60-80	55	III	No	Yes	12	18	91
id9	30	Married	Univ	SL	>80	37	III	Yes	No	15	19	97
id10	50	Other	Coll	SL	40-60	32	II	Yes	No	17	18	100
id11	52	Married	Coll	PTW	20<	40	I	Yes	Yes	22	13	32
id12	47	Single	Univ	SL	40-60	14	DK	Yes	No	11	17	91
id13	29	Single	Colle	FTW	>80	21	II	No	No	9	16	88
id14	37	Divorced	Coll	SL	20<	28	II	No	No	11	19	94
id15	45	Divorced	Univ	SL	40-60	41	II	No	No	18	18	94
id16	60	Other	Coll	SL	20-40	74	II	No	No	11	21	38

*Note*. DK = Don’t know; Univ = University; Coll = College; High S = High school; SL = Sick leave; FTW = Full-time work; PTW = Part-time work; BMI = Body Mass Index; PHQ = Patient Health Questionnaire; GAD = General Anxiety Disorder; Adherence = Number of adapted physical activity sessions realized.

^a^Chemotherapy or radiotherapy.

### Intervention Effects

**Figure 2:**
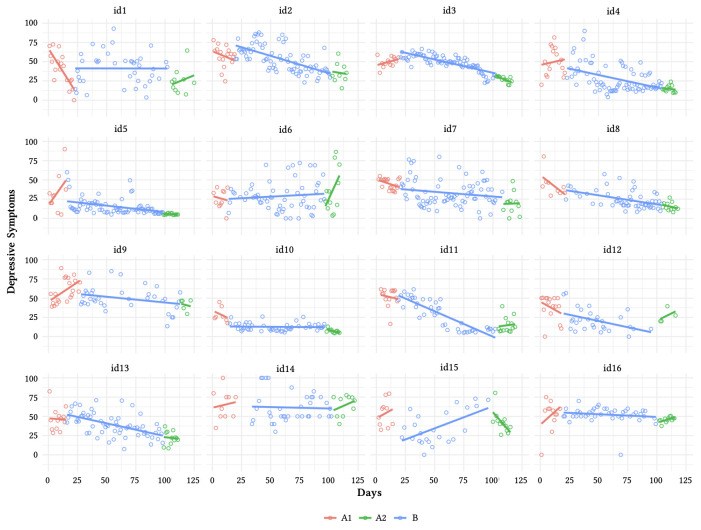
Depression Scores in Three Conditions With Regression Lines for Each Phase

**Figure 3:**
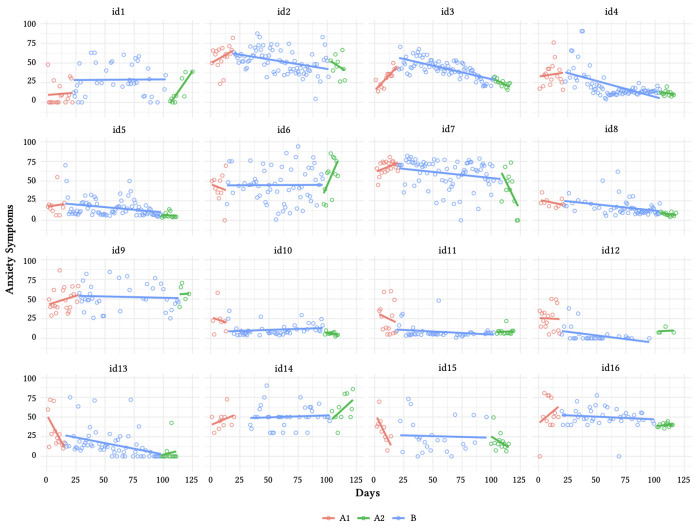
Anxiety Scores in Three Conditions With Regression Lines for Each Phase

Figures 2 and 3 present the daily mean of depression and anxiety symptoms for all participants and local regressions. Table 3 and 4 present the results of piecewise regressions. The level regression coefficient indicates a mean level change after the beginning of intervention. The slope regression coefficient indicates the daily decrement following the beginning of intervention.

**Table 3:** Results of Piecewise Regressions for Depressive Symptoms

ID	Phase B vs A						Phase A2 vs A					
	Level			Slope			Level			Slope		
	*B*	2.5%	97.5%	*B*	2.5%	97.5%	*B*	2.5%	97.5%	*B*	2.5%	97.5%
1	14.36	-9.73	38.45	1.19	-.06	2.45	68.32	-27.02	163.66	3.14	-1.04	7.32
2	15.27*	4.81*	25.73*	-.48*	-.63*	-.33*	-16.67	-37.32	3.98	-.88	-4.46	2.71
3	9.62*	2.48*	16.76*	-.30*	-.43*	-.17*	-17.86*	-29.59*	-6.14*	-.58	-1.47	.31
4	-.40*	-.56*	-.23*	-.03*	-.04*	-.02*	-3.38*	-4.45*	-2.32*	-.03	-.07	.01
5	-.66*	-.84*	-.48*	-.08*	-.09*	-.05*	-7.46*	-9.26*	-5.65*	-.05*	-.09*	-.01*
6	.40	-18.73	19.53	.15	-1.13	1.42	-10.76	-131.59	110.07	3.37*	.98*	5.76*
7	-9.99	-22.93	2.94	-.08	-.27	.11	-32.09*	-55.81*	-8.37*	.30	-3.42	4.02
8	-.17*	-.32*	-.03*	-.01*	-.01*	-.01*	-.94*	-1.21*	-.68*	-.01	-.03	.01
9	-3.60	-13.23	6.04	-.52	-1.67	.62	-41.06	-95.19	13.06	1.84	-7.65	11.33
10	-15.81*	-20.70*	-10.91*	-.01	-.08	.06	-19.95*	-26.85*	-13.05*	-.21	-.68	.26
11	2.26	-5.91	10.42	-.86*	-1.03*	-.69*	-39.12*	-51.51*	-26.73*	.39	-.94	1.72
12	-12.99*	-16.80*	-9.18*	-.36*	-.50*	-.22*	-20.94*	-38.72*	-3.17*	4.35	-7.43	16.14
13	7.00*	.58*	13.43*	-.35*	-.45*	-.26*	-22.92*	-33.91*	-11.93*	-.07	-1.44	1.29
14	-2.59	-19.37	14.18	-.08	-.31	.15	2.19	-23.44	27.81	-.49	-2.71	1.74
15	-23.85*	-43.17*	-4.53*	.47*	-.05	1.00	3.10	-18.50	24.70	-.96	-2.26	.34
16	1.29	-12.16	14.73	-.25	-1.28	.79	-25.21	-118.30	67.88	.31	-1.22	1.84

*p < .05.

**Table 4:** Results of Piecewise Regressions for Anxiety Symptoms

ID	Phase B vs A						Phase A2 vs A					
	Level			Slope			Level			Slope		
	*B*	2.5%	97.5%	*B*	2.5%	97.5%	*B*	2.5%	97.5%	*B*	2.5%	97.5%
1	7.70	-7.68	23.08	-.96	-1.70	-.21	-80.52	-138.72	-22.33	2.20	-.87	5.28
2	2.81	-8.38	13.99	-.23*	-.41*	-.06*	-8.18	-28.24	11.88	-1.03	-4.22	2.16
3	19.86*	10.50*	29.23*	-.27*	-.41*	-.13*	-2.47	-17.16	12.23	-.77	-2.02	.49
4	-.16	-.35	.03	-.05*	-.06*	-.04*	-3.98*	-5.23*	-2.73*	-.05*	-.09*	.00*
5	-.10	-.32	.12	-.06*	-.08*	-.03*	-5.48*	-7.63*	-3.32*	-.04*	-.08*	.00*
6	6.80	-5.74	19.33	-.03	-.20	.15	-8.73	-28.43	10.96	3.38*	1.45*	5.31*
7	-2.56	-13.39	8.26	-.14	-.30	.02	-11.71	-33.53	10.11	-3.75*	-7.27*	-.23*
8	4.84	-4.43	14.11	-.01	-.30	.27	.81	-35.27	36.90	.01	-.49	.51
9	6.78	-2.22	15.78	-.89	-1.92	.14	-40.63	-89.36	8.11	3.83	-5.02	12.68
10	-17.71*	-24.55*	-10.88*	-.17	-.68	.33	-37.82	-83.46	7.82	-.28	-.95	.40
11	-11.82*	-19.83*	-3.81*	.43	-.46	1.33	20.40	-44.40	85.20	.41	-.74	1.56
12	-21.19*	-27.05*	-15.34*	-.72*	-1.23*	-.20*	-45.22*	-85.39*	-5.06*	-1.19	-11.24	8.86
13	.06	-.20	.09	-.02*	-.02*	-.02*	2.57*	-3.22*	-1.99*	.08*	.01*	.15*
14	4.94	-6.39	16.28	-.02	-.17	.13	1.24	-17.48	19.97	1.44	-.25	3.13
15	-10.21	-22.74	2.32	-.14	-.46	.17	-9.41	-23.57	4.76	-.75	-1.85	.34
16	-.79	-8.00	6.41	-.08	-.20	.04	-14.47	-28.91	-.04	.08	-1.48	1.64

*p < .05.

A significant decrease of depressive symptoms during the intervention has been found among 10 participants (id2, id3, id4, id5, id8, id13, id10, id11, id12, id15, see details in Table 2). As presented in Figure 2, different patterns of symptom reduction can be observed. For id4, id5, id8, id12, a negative and significant effect was found for level and slope. In other words, benefits from our intervention were quick and progressive. For id2, id3, id13, the beginning of intervention was associated with a significant higher level of symptoms, however, these symptoms gradually decreased (i.e., negative slope) during the intervention. A significant reduction of daily depressive symptoms was observed only for the level (id10, id15) or slope (id11).

A significant decrease of anxiety symptoms following the introduction of the intervention has been found for nine participants (id1, id2, id3, id4, id5, id10, id11, id12, id13, see details in Table 3). Different patterns of symptom reduction have also been found (see Figure 3). For id12, a negative and significant effect was found for level and slope. For id3, the beginning of intervention was associated with significant a higher level of anxiety symptoms, however, these symptoms gradually decreased during the intervention. A significant reduction of daily anxiety symptoms was observed only for the level or slope for id10, id11, and id1, id2, id4, id5, id13, respectively.

In summary, a significant reduction of depression and anxiety symptoms was found among seven participants (id2, id3, id4, id5, id10, id12, id13). A no significant effect of our intervention on mental health symptoms was found for four participants (id6, id9, id14, id16). Also, id6 showed a significant progressive increase of depressive and anxiety symptoms during the follow-up phase.

Significant slope changes were observed more often than level changes (8/10 for depressive and 7/9 anxiety symptoms), indicating that the impact of PA on mental health was generally gradual. Seven participants with a significant reduction of depressive symptoms during the intervention showed maintained benefits during the follow-up phase (id, 3, id4, id5, id8, id11, id12, id13). Four women experienced maintained benefits in terms of anxiety (id1, id4, id5, id13). The statistical modeling of time series explained 15% to 74%, and 12% to 54% of the variance for depressive and anxiety symptoms, respectively (more details are provided in the Supplementary Materials [(Bernard, 2025S]).

A visual analysis of repeated PHQ-9 and GAD-7 scores (see Figure S.1. and S.2. in the Supplementary Materials [Bernard, 2024S-b]) shows that most of the participants (with complete data) had a score below the clinical cut-off at the end of intervention.

### Personal Event During Intervention

Eleven participants were available for the short interview at the end of intervention. The reported personal events are presented in the Supplementary Materials (Bernard, 2025S).

## Discussion

The primary aim of this study was to evaluate the short-term effects a 12-week remote adapted PA intervention on daily levels of depressive and anxiety symptoms among women with BC and severe depressive or anxiety symptoms. We hypothesized that daily level of depressive and anxiety symptoms would decrease during the intervention and that these benefits would be maintained two weeks after intervention. Our findings show that our program progressively decreases the depressive and anxiety symptoms among 10 and nine participants, respectively. This significant decrease of symptoms could be considered as clinically relevant because observed benefits were corroborated with the decrease PHQ-9 and GAD-7 after the intervention. After Phase B, most of our patients showed improvement, moving from a severe to a moderately severe or moderate depression score, and from a moderately severe to a moderate anxiety score. Overall, this study supports the utility of remote PA intervention to improve depressive and anxiety symptoms in women with BC and poor mental health. It is difficult to compare our results to previous studies as no other study has examined the effect of PA in this specific population. However, our positive findings are in line with previous meta-analyses suggesting significant benefits of physical activity on mental health in women with breast cancer (Carayol et al., 2013, 2015).

The remote format of our intervention is particularly promising because physical distance from care and the disproportionate distribution of mental healthcare providers are two major barriers for patients with cancer and severe psychological distress (Deshields et al., 2021). Another important finding is that the benefits from PA were generally not immediate, i.e., not associated with the beginning of intervention. It is in line with the Canadian treatment guidelines for mood disorders concluding that PA interventions length has to be superior to 10 weeks to decrease the depression severity (Ravindran et al., 2016). This progressive effect is also in line with previous n-of-1 studies examining the benefits of PA in young adults with high depressive symptoms (McFadden et al., 2017) or women with major depressive disorders (Doyne et al., 1983).

Our intervention did not show significant effect for all included women. The absence of mental health benefits from our intervention could be partially explained by personal events during the intervention: cancer treatment (id16) or mourning experiences (id14). Other factors could also be associated with responsiveness to the intervention such as a low adherence rate (e.g., id16), intensity of PA session (Bernard et al., 2013), or type of exercise. Indeed, Carayol et al. (2015) suggested that yoga related activities led to greater decrease of depressive and anxiety symptoms rather than aerobic/resistance-PA during BC treatment. The fear of cancer recurrence (Savard & Ivers, 2013) might also play a moderating role in the effectiveness of PA intervention for this population. Future studies should consider this factor as a judgment or inclusion criterion to optimize the benefits of PA program.

To our knowledge, this is the first study examining the effects of a PA intervention among women with BC and severe depressive or anxiety symptoms. Although this study employed a robust method, our results have to be replicated with a larger sample to understand the PA intervention response heterogeneity. Also, more complex models should also be used to examine the daily dynamic of mental health outcomes (Batley, 2024).

While our results are promising, this study is not without limitations. Firstly, repeatedly asking study participants about their mental health symptoms with EMA may increase their awareness about them (Runyan et al., 2013), thus influence outcomes and threat the internal validity in our N-of-1. Secondly, women with metastatic BC were excluded from our study because we could not fully guarantee the safety of these patients during remote PA sessions. Future studies should investigate how to implement a safe remote PA intervention for this population, who is less aerobically fit, more symptomatic and report higher levels of fatigue and dyspnea (Yee et al., 2014). Thirdly, the collection of information regarding impactful life events that could influence our result was missing for 4 participants. Consequently, we could not check the internal validity our findings.

In conclusion, the present study is the first to report on the short-term effect of remote adapted PA for the treatment of severe depressive or anxiety symptoms. Future investigations could assess the efficacy of several strategies to improve the maintenance of mental health benefits, such as offering additional booster sessions as needed. Our findings are relevant for clinical practice, as they suggest that our intervention can be easily implemented for women with BC living far to psycho-oncology services.

## Supplementary Materials

The Supplementary Materials contain the following items:

- Preregistration (Bernard et al., 2022S)
- Research data and R code (Bernard, 2024S-a)
- Additional figures (Bernard, 2024S-b)
- Additional information:
  - Detailed statistic findings (Bernard, 2025S)
  - SCRIBE checklist (Bernard, 2025S)
  - Interview findings (Bernard, 2025S)

BernardP.
 (2024S-a). A remote adapted physical activity intervention for women with breast cancer and severe depressive or anxiety symptoms: Series of N-of-1 trials with ecological momentary assessment: DATA + R code
[Research data and R code]. PsychOpen. https://osf.io/m7xpu/
10.32872/cpe.14851PMC1292318641727814

BernardP.
 (2024S-b). A remote adapted physical activity intervention for women with breast cancer and severe depressive or anxiety symptoms: Series of N-of-1 trials with ecological momentary assessment: Figures
[Additional figures]. PsychOpen. https://osf.io/z7tm5/
10.32872/cpe.14851PMC1292318641727814

BernardP.
 (2025S). A remote adapted physical activity intervention for women with breast cancer and severe depressive or anxiety symptoms: Series of N-of-1 trials with ecological momentary assessment: Supplementary files
[Additional information]. PsychOpen. https://osf.io/tmfub/
10.32872/cpe.14851PMC1292318641727814

BernardP.
LapointeJ.
CaudroitJ.
ComtoisA. S.
LanovazM. J.
 (2022S). Effect of an adapted physical activity intervention in women with breast cancer and depressive or anxiety symptoms: A set of single case experimental studies combined with ecological momentary assessments
[Preregistration]. PsychOpen. 10.17605/OSF.IO/9SBJH


## Data Availability

Data, open materials, and R scripts are available online (see Bernard, 2024S-a, 2024S-b, 2025S).

## Social Media Accounts

*Paquito Bernard:* Mastodon, Bluesky

## Preregistration

The study protocol was registered a priori (see Bernard et al., 2022S).

## Reporting Guidelines

This manuscript was written according to the Single-Case Reporting Guideline in BEhavioural Interventions.
